# Comparison of adhesiveness between new improved surgical drapes and existing drapes during contraceptive surgery in 90 cats: a multi-center controlled clinical trial

**DOI:** 10.3389/fvets.2026.1667777

**Published:** 2026-08-11

**Authors:** Ryosuke Namiki, Akiko Uemura, Kazumi Shimada, Takashi Tanaka, Tomohiko Yoshida, Akitaka Onitsuka, Ahmed Mandour, Shingo Ogawa, Ryou Tanaka

**Affiliations:** 1 Department of Veterinary Medicine, Tokyo University of Agriculture and Technology, Fuchu, Tokyo, Japan; 2 Department of Clinical Veterinary Medicine, Obihiro University of Agriculture and Veterinary Medicine, Obihiro, Hokkaido, Japan; 3 Cosmotec Co., Ltd., Tachikawa, Tokyo, Japan; 4 Department of Veterinary Internal Medicine, Suez Canal Universit, Ismailia, Egypt; 5 Dainichiseika Colour and Chemicals Mfg. Co., Ltd., Chuouku, Tokyo, Japan

**Keywords:** base film, flexibility, plastic drape, structure, surgical site infection

## Abstract

Plastic drapes are used prevent surgical site infections by enclosing bacteria within adhesive layers that directly contact the skin during surgery. However, these drapes often peel from the surgical edge, increasing the risk of surgical site infection. This problem has remained unresolved since the development of plastic drapes decades ago. Recently, a basic study using rat skin reported improvements in the structure of these drapes. This study indicated that drape detachment during skin incision can be suppressed by improving the flexibility of the drape base film. Furthermore, although the number of cases was small, a clinical study was conducted, and the improved flexible drape was found to peel off less during cat surgery. The purpose of this study was to compare the peeling area of an existing drape with that of a prototype drape with an improved flexible base film in a large number of feline cases as part of a multi-center controlled clinical trial. Ninety cats that underwent neutering were randomly assigned to the existing drape group and the prototype drape group. Immediately after the skin incision and before suturing, the surgeon graded the degree of detachment of the drape from the surgical edge based on the diagram prepared in advance. The peeling grade of the prototype drape was lower than that of the existing drape at both times. We concluded that the prototype drape with a more flexible base film could reduce the intraoperative drape peeling area.

## Introduction

Surgical site infection (SSI) is defined as an infection that occurs within 30 days after surgery in human medicine (1, 2). Unfortunately, there is no exact definition of SSI in veterinary medicine to date. In any case, SSI not only threatens the lives of patients but also increases physical and economic burden (3). SSI is considered one of the undesirable complications that can occur after surgery. The pathogens that cause SSI are mainly skin-resident flora such as *Staphylococcus pseudintermedius* (4, 5). It is thought that SSI occurs when these skin-resident bacteria horizontally invade the surgical wound during surgery (6).

**Figure 1:**
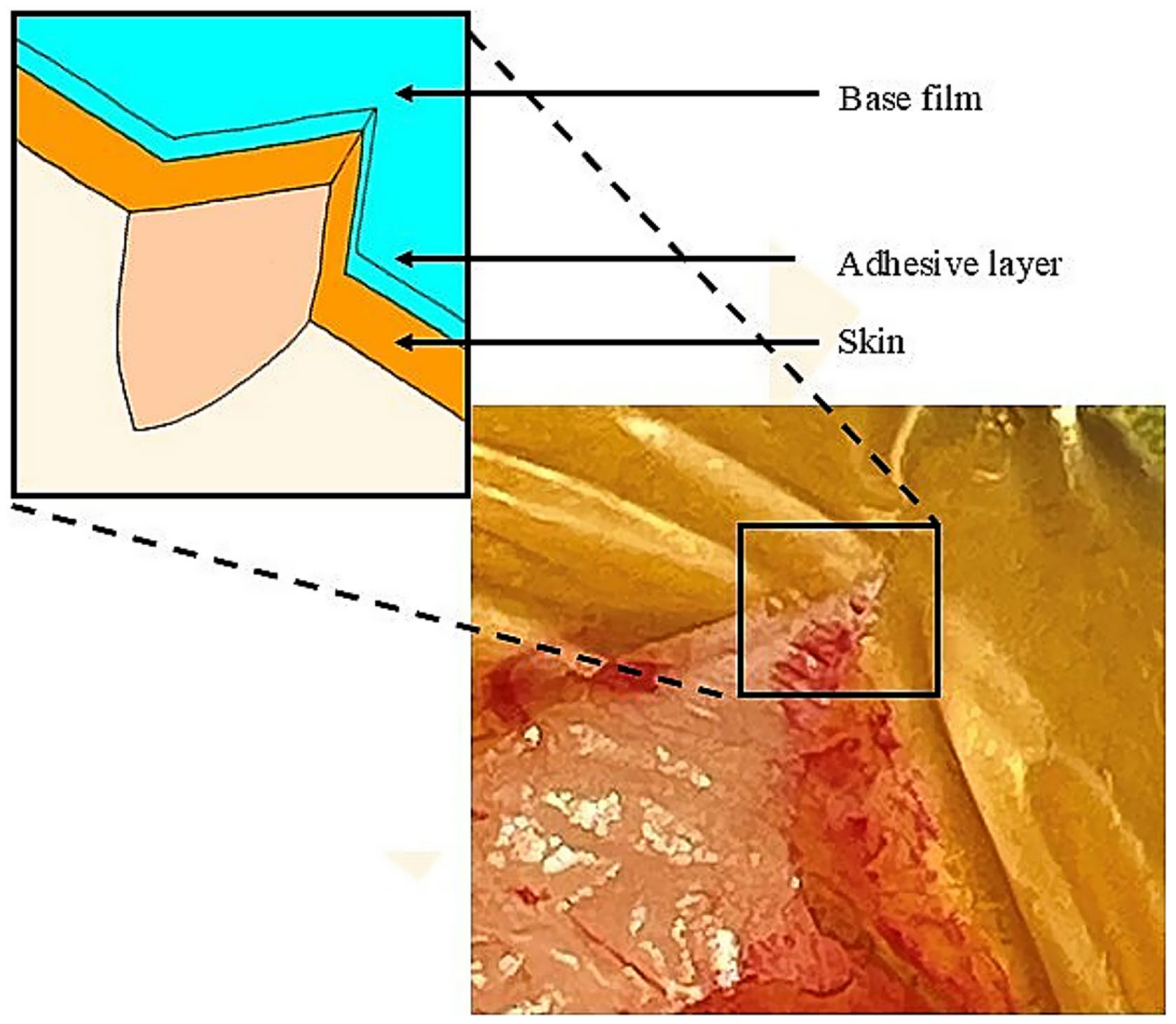
Structure of plastic drapes. The photo on the bottom right shows the drape attached to the skin incision margins. The image on the top left shows a close-up of the drape structure. The plastic drape consists of an adhesive layer applied directly to the skin and a base film that supports the adhesive layer. The adhesive layer encloses skin flora within the layer, preventing them from migrating to the surgical field during surgery.

Plastic drapes have been used for decades to prevent surgical site infections (SSIs) in human and veterinary medicine across various procedures, including chest, abdominal, neurological, orthopedic, and ophthalmic surgeries (7). The structure of plastic drapes has remained largely unchanged from their early days to the present, and it consists of two layers: a base film made of polyurethane or polypropylene and an adhesive layer that is applied directly to the skin (Figure 1). The adhesive layer of the plastic drape is attached to the skin to enclose bacteria in the adhesive layer, preventing them from migrating from the wound edge to the surgical field during surgery (8, 9). In human medicine, it has been reported that the use of drapes with good adhesiveness reduces the incidence of SSIs and improves the overall cost-effectiveness (3, 10). In recent years, a series of studies have been published suggesting the usefulness of isodine-impregnated plastic drapes in preventing infection during surgery (2, 8, 11–14). In veterinary medicine, a clinical study on horses has reported that surgeries performed with plastic drapes reduced wound complications compared to those without drapes (15). Therefore, plastic drapes are clinically useful for preventing SSIs, and their use is expected to increase worldwide in the future (16).

Although plastic drapes have many advantages, some problems have been identified. One of these problems is that plastic drapes frequently peel off from the wound edge during surgery in human and veterinary medicine (17–19). In human medicine, it has been reported that drape peelings increase the risk of postoperative infection (9, 20, 21). Of course, when a plastic drape peels off, it cannot prevent SSI or foreign bodies from entering the wound (22). Furthermore, the drape that peels off from the wound would obstruct the view of surgeons. Therefore, in order for plastic drapes to fully perform their functions, they must be in close contact with the surgical field throughout the whole operation.

It is necessary to improve the adhesion of the drape to the skin so that the drape can adhere closely to the wound edge. The focus was on improving the structural conditions of the drape to achieve this purpose. Plastic drapes are a type of adhesive with a two-layer structure. Generally, the adhesive strength of the adhesive changes depending on several factors, such as the width, thickness, and elasticity of the adhesive layer and the base film (23). Previous research reported that thickening the adhesive layer of the drape increased the adhesive strength and suppressed drape peeling during skin incision in rats (24). After that, another study found that increasing the flexibility of the base film (i.e., lowering the Young’s modulus of the base film) reduced drape peeling during skin incision in rats (25, 26). Furthermore, it has been reported that the prototype drapes with a thicker adhesive layer and a more flexible base film reduced the drape peeling area during surgery in cats compared to the existing drapes (27).

However, this study was limited to a small number of cases by one surgeon at a single institution. In addition, as the thickness of the plastic drape increases, it is expected that the drape will become more difficult to cut than the existing drapes. Therefore, in this study, we developed a new prototype drape with a more flexible base film while maintaining an adhesive layer thickness almost the same as the existing drapes. We increased the number of cases at multiple institutions and conducted a randomized controlled clinical study.

The purpose of this study was to develop a prototype drape with improved flexibility of the base film and to examine whether the peeling issue during actual surgery would improve compared to cases where existing drapes were used.

## Materials and methods

The study was a multi-center controlled clinical trial involving five veterinary practices in Japan. All experimental procedures and protocols were approved by the Tokyo University of Agriculture and Technology (TUAT, Approval number: 30-64). All owners had given their written informed consent before the start of the study.

### Animals

Client-owned cats of any breed, age, and body weight that underwent neutered surgery could enroll in the study. Exclusion criteria included cases involving an anesthesia duration of 60 min or longer and cases with excessive intraoperative bleeding.

### Plastic drapes

**Figure 2:**
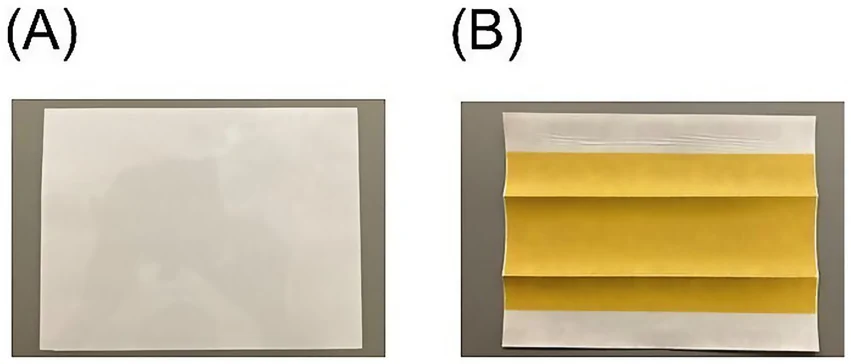
Plastic drapes were used in this study: the prototype drape **(A)** and the existing drape **(B)**. The existing drape was a commercially available product called 3M™Ioban™Incise drape® that was impregnated with iodine.

In this study, we used two plastic drapes: the prototype and the existing plastic drapes (Figure 2; Table 1).

**Table 1:** Specification of two types of drapes.

Drape Type	Thickness of adhesive layer	Young’s modulus of the base film
Prototype drape	20 μm	1.78 MPa
Existing drape	25–30 μm	18.00 MPa

The prototype drape was set to have approximately the same thickness as the existing drape, while the base film of the prototype drape was more flexible than that of the existing drape.

The prototype plastic drape also had two layers: the base film and the adhesive layer. The adhesive layer was made of acrylic, and the thickness was set to 20 μm. The base film was made of a urethane sheet, and Young’s modulus was measured to be 1.78 MPa.

3M™Ioban™Incise drape® (3M Japan Co., Ltd., Tokyo, Japan) was used as the existing drape. The adhesive layer was made of acrylic, and the thickness was measured to be 25–30 μm. The base film was made of a polyester sheet, and Young’s modulus was measured to be 18.00 MPa. The color of these drapes is yellow.

The size of both drapes was 20 cm × 10 cm. All drapes were sterilized by gamma irradiation and were stored at room temperature. The existing drapes were within their expiration date. The prototype drapes were made just before the start of this study. The color of these drapes was clear.

### Study design

Ninety cats were randomly allocated to one of two groups: the prototype drape group (*n* = 45) and the existing drape group (*n* = 45). The randomization method used was simple randomization; specifically, the drapes were assigned alternately at each clinic based on the order in which the surgeries were performed. In each clinic, only one surgeon was selected to perform the procedures.

### Preparation of surgical fields and operation

**Figure 3:**
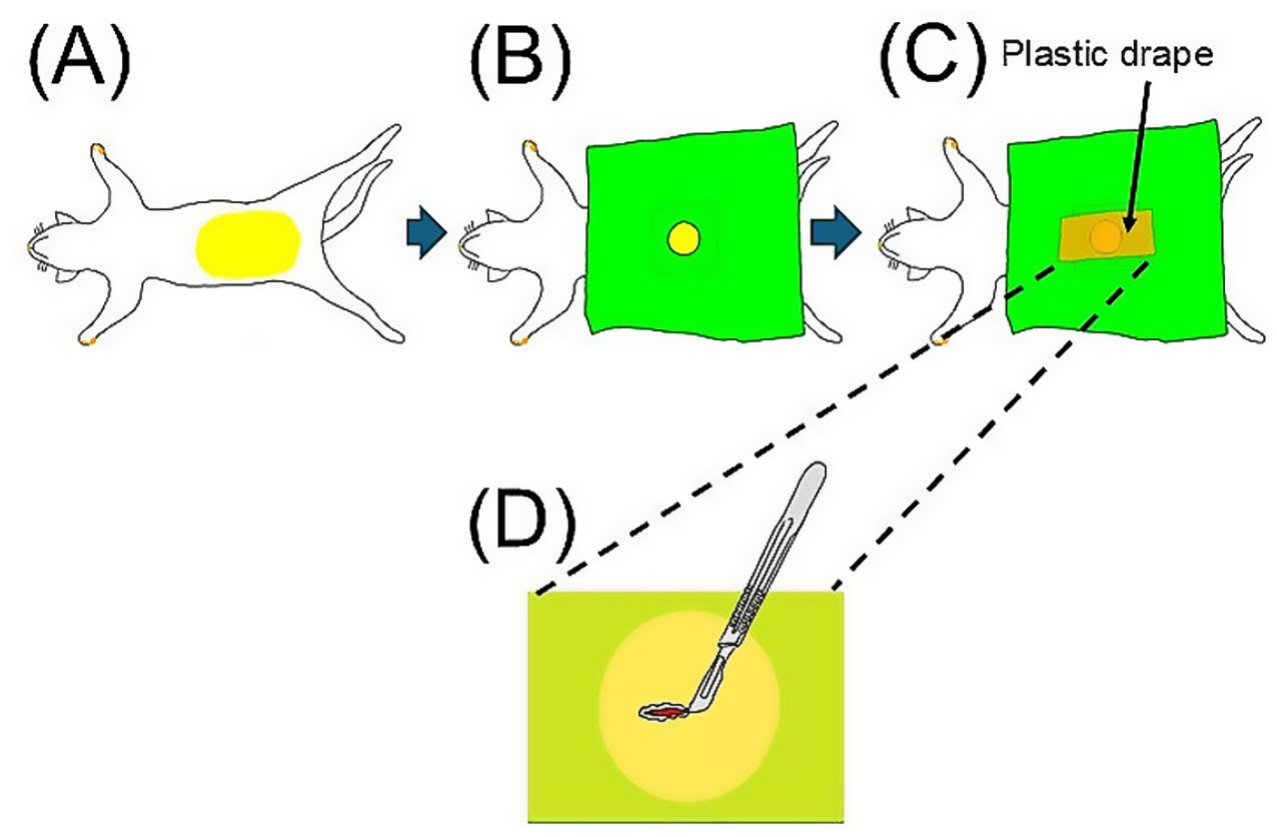
Preparation of the surgical field. Cats were laid in the supine position, and the hair on the lower abdomen was cut using clippers **(A)**. The clipped area was scrubbed twice with chlorhexidine using the concentric circular scrub method. Thereafter, gauze soaked in chlorhexidine alcohol was used to disinfect the area twice. The non-adherent cloth drape with a circular window with a diameter of 9 cm was applied to the surgical area **(B)**. The surgeon and an assistant applied the plastic drape over the cloth drape **(C)**. The surgeon cut the plastic drape and the skin simultaneously with a scalpel **(D)**.

All cats included in the study received routine general anesthesia with endotracheal intubation at their respective clinics. Cats were laid in the supine position. The hair on the lower abdomen of cats was cut using clippers so that the hair did not extend into the 9 cm diameter cloth drape window (Figure 3A). The clipped area was scrubbed twice with chlorhexidine acetate (Nolvasan Surgical Scrub, Zoetis Inc., Madison, NJ, United States) using the concentric circular scrub method. Thereafter, gauze soaked with chlorhexidine alcohol (0.5% Hexizac Alcohol Solution N, Yoshida Pharmaceutical Co. Ltd., Tokyo, Japan) was used to disinfect the area twice. The chlorhexidine alcohol was thoroughly evaporated for at least 1 min before applying the drape. The above preparation methods and the drugs to be used in the surgical field were instructed at each clinic in advance. The non-adherent cloth drape with a circular window with a diameter of 9 cm was applied to the surgical area (Nagai Leben Co. Ltd., Tokyo, Japan) because the plastic drapes could not cover the whole body (Figure 3B). The surgeon and the assistant applied the plastic drape over the cloth drape (Figure 3C). The surgeon cut the plastic drape and the skin simultaneously with a No. 15 scalpel (ELP surgical blade, Akiyama Medical MFG. Co. Ltd., Tokyo, Japan) (Figure 3D). After laparotomy, spay surgery was performed in the usual way. The surgical procedure for spay surgery consisted of removal of the ovaries and uterus. The drape was removed by hand before the skin was sutured.

### The questionnaire

The surgeon answered the questionnaire regarding the date of surgery, the duration of surgery, the extent of bleeding, the drape breakage, and the condition of the skin of the surgical field where the drape was applied. The duration of surgery was recorded by the surgeon on a three-level scale: less than 30 min, 30 to 60 min, and more than 60 min. The skin of the surgical field where the drape was applied was observed macroscopically for swelling, color change, and epithelial delamination to assess harm to the skin caused by the drape. In addition, the surgeons responded to whether there were any differences between the two groups in terms of the ease of cutting the drape when making the incision and the ease of removing the drape from the skin after surgery.

### The assessment of the drape peeling area

**Figure 4:**
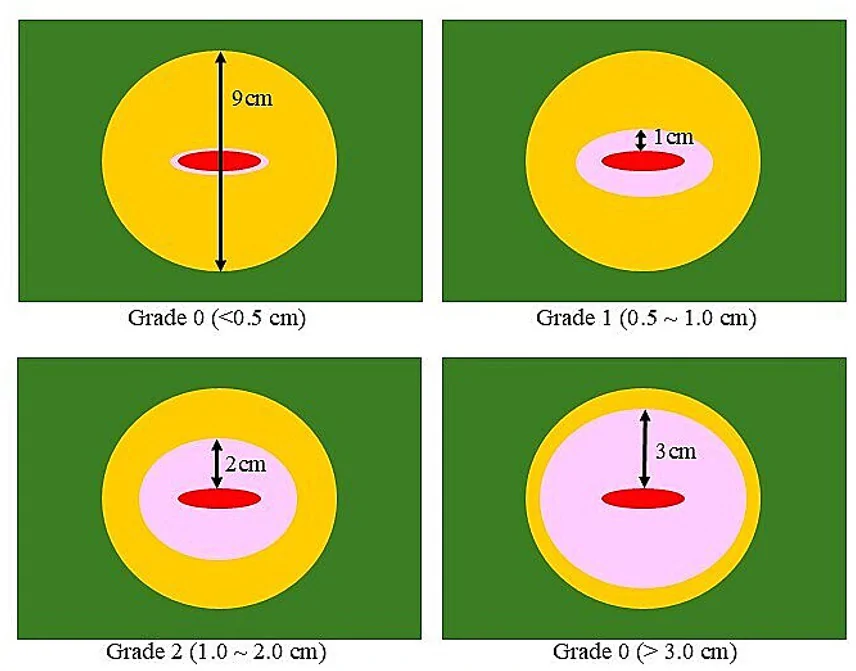
Drape peeling grade diagram. Immediately after skin incision and before skin suture, the surgeon graded the drape peeling area based on this diagram. The grade was divided into four stages according to the length of detachment from the surgical edge. The diagram that the surgeon recognized as the most similar was determined as the drape peeling grade.

Immediately after skin incision and before skin suture, the surgeon evaluated and graded the drape peeling area based on the drape peeling grading diagram subjectively (Figure 4). The grade was divided into four stages according to the length of detachment from the surgical edge, as shown in the figure. The diagram that the surgeon recognized as the most similar was determined as the drape peeling grade.

### Statistical analyses

Data analysis was conducted using a commercial statistics software package (GraphPad Prism Version 8.0, GraphPad Software Inc., La Jolla, CA, United States). *p* < 0.05 was considered statistically significant. Because the adhesion score was an ordinal variable, comparisons between both groups at each time point were performed using the Mann–Whitney *U*-test. A cumulative linked mixed model including cat as a random effect was explored to account for repeated measurements; however, the model failed to converge because of sparse observations in the highest score category and quasi-complete separation. Therefore, non-parametric analysis was retained.

## Results

Ninety female cats that underwent neutering surgery from December 2022 to June 2023 were used in this study.

There were eight cases in the prototype drape group and six cases in the existing drape group, with a surgery duration of 30–60 min. No cases had an operation time exceeding 60 min. All surgeries were finished without excessive bleeding or anesthesia-related problems. As a result, all female cats that underwent spay surgery at five clinics during this period were included in this study.

There were no cases of grade 3 drape peeling in either group (Table 2).

**Table 2:** Drape peeling grade distribution.

Grade	Prototype drape		Existing drape	
	Immediately after skin incision	Before skin suture	Immediately after skin incision	Before skin suture
0	44	41	38	21
1	1	4	7	22
2	0	0	0	2
3	0	0	0	0

Large-scale peeling suggesting grade 3 was not observed in either group. In the existing drape group, two cases of grade 2 peeling were observed before skin suturing.

The drape peeling grade immediately after skin incision was significantly lower in the prototype drape group (0.025 ± 0.149) than in the existing drape group (0.160 ± 0.367) (*p* = 0.03).

The drape peeling grade increased with the course of surgery in both groups.

**Figure 5:**
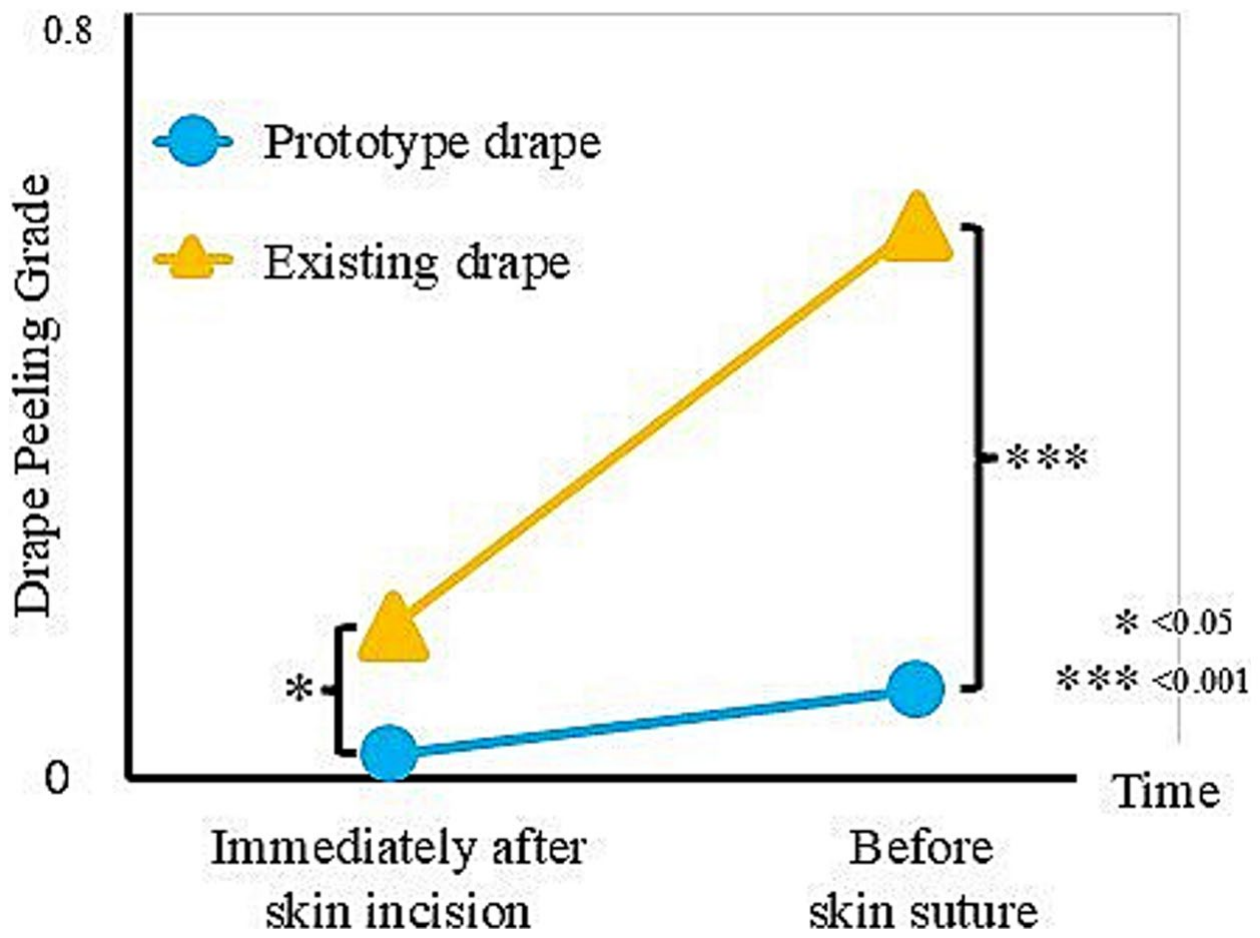
Drape peeling grades in both groups. The drape peeling grades at both times were significantly lower in the prototype drape group than in the existing drape group. The drape peeling grade increased with the course of surgery in both groups.

The drape peeling grade before skin suture was significantly lower in the prototype drape group (0.093 ± 0.287) than in the existing drape group (0.578 ± 0.583) (*p* = 0.00000736) (Figure 5; Table 3).

**Table 3:** Drape peeling grade.

Drape type	Immediately after skin incision	Before skin suture
Prototype drape	0.025 ± 0.149	0.093 ± 0.287
Existing drape	0.160 ± 0.367	0.578 ± 0.583

The drape peeling grade was significantly lower for the prototype drape than for the existing drape both immediately after skin incision and before skin suture. The difference was greater immediately after skin incision and before skin suture.

In the questionnaire for surgeons, there were no reports of notable breakage or tearing of the drapes. The drape-related skin disorders were reported as minor, such as temporary redness (one case in the prototype group and one case in the existing group). No differences were reported between the two groups in terms of ease of cutting the drape during incision or ease of removing the drape from the skin after surgery.

## Discussion

Plastic drapes are adhesive, and the thicker the adhesive layer, the stronger the adhesive strength (23, 28). In fact, a report found that a prototype drape with a thicker adhesive successfully reduced the peeling area in rat surgery (24). However, a thicker adhesive layer requires extra force when cutting the drape with a scalpel during skin incision. Additionally, a thicker adhesive layer reduces shearing force, which can cause the contact surface of the skin and the adhesive layer to shift horizontally when a scalpel is moved during skin incision (24, 29). Furthermore, when the adhesive layer is excessively thick, during removal of the drape at the end of surgery, the drape may not peel off at the surface between the drape and the skin but may be peeled off inside the adhesive layer (i.e., cohesive failure). In cases where cohesive failure occurs, part of the adhesive layer may remain on the skin for a long time; it is not only unsanitary but also increases the possibility of causing contact dermatitis (30–32). Considering these points, in this study, the thickness of the adhesive layer of the prototype drape was set to be almost the same as that of the existing drape. The questionnaire survey of surgeons revealed no difference between the two groups, including the ease of cutting during skin incision.

**Figure 6:**
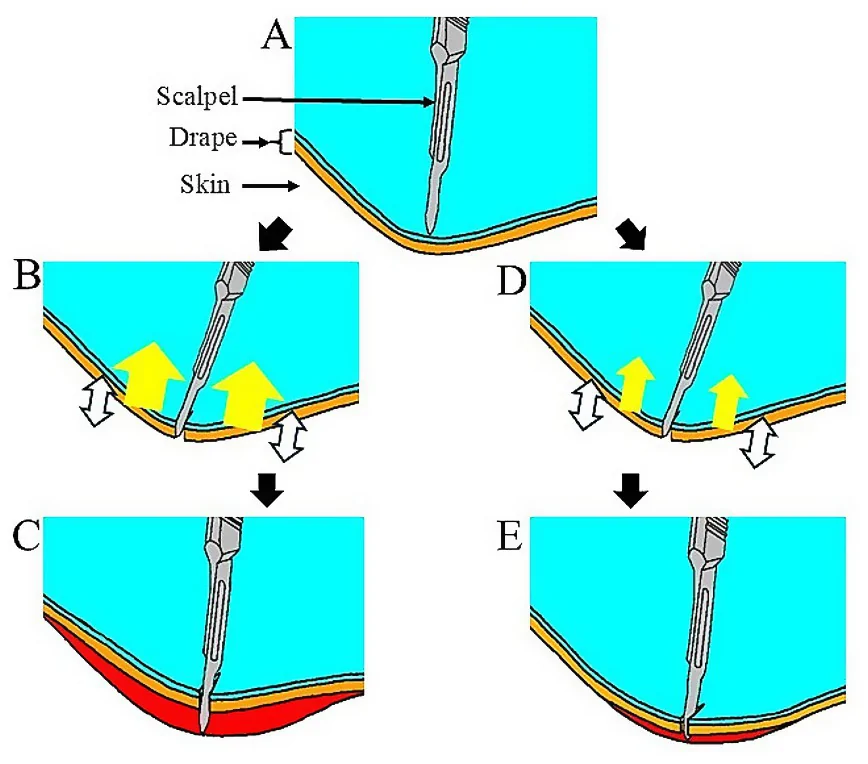
Separation mechanism of drapes during the incision of the skin. The surgeon pushes the drape and the skin together with a scalpel. The scalpel presses down on the drape, causing it to distort downward **(A)**. Subsequently, when the scalpel breaks through the drape before the skin is cut, the drape is in close contact with the skin because of adhesive strength (white arrow). Drape tries to return upward because upward stress (yellow arrow) is generated in the distorted drape **(B)**. The drape and skin peel off when the stress generated in the drape surpasses the adhesive strength between the drape and the skin **(C)**. The upward stress generated within the drape will be smaller for the drape with a flexible base film (yellow arrow) **(D)**, and the flexible drape with the lowest stress generated within it will be less likely to peel off from the skin, resulting in a smaller drape peeling area (red area) **(E)**.

The results showed no major peeling of grade 3 or drape, but peeling cases up to grade 2 were observed in both drape groups. The separation of drapes during skin incision is thought to occur through the following mechanism. First, the surgeon pushes down the drape and the skin together with a scalpel. The scalpel presses down on the drape, causing it to distort downward (Figure 6A). Subsequently, the scalpel breaks through the drape before the skin is cut. At this moment, the drape is in close contact with the skin because of the adhesive strength (white arrow). But the drape tries to return upward because upward stress (yellow arrow) is generated in the distorted drape (Figure 6B). Shortly after that, the drape and skin peel off when the stress generated in the drape surpasses the adhesive strength between the drape and the skin (Figure 6C). The drape peeling during skin incision was thought to occur due to this mechanism described above in this study.

The relationship between Young’s modulus, stress, and strain can be expressed by the following formula:

$$\text{Stress}[\sigma ]=\text{Youn}g^{\prime }s\;\text{modulus}[E]\times \text{strain}[\varepsilon ]$$

Therefore, assuming that the downward strain [*ε*] caused by the scalpel during skin incision is the same for both groups, the upward stress [*σ*] (yellow arrow) generated within the drape will be smaller for the drape with a base film with a lower Young’s modulus [*E*] (i.e., a flexible base film) (Figure 6D) (25). When the adhesive strength of the drape is the same, the prototype drape with the lower stress generated within it was thought to be less likely to peel off from the skin, resulting in a smaller drape peeling area (Figure 6E).

In both groups, the drapes became increasingly detached from the skin as surgery progressed. The drape peeling during surgery was thought to be caused by two reasons. First, peeling is caused by traction of the skin from the surgeon’s surgical manipulation. Another reason is moisture between the adhesive layer of the drape and the skin. Generally, the adhesive strength is reduced by the moisture between the adhesive and the adherend. It has been reported that moisture in the surgical field can enter between the skin and the drape, causing them to separate over time (33). In this study, the surgeon’s manipulation and the moisture from bleeding and exudates during surgery gradually penetrated between the drape and the skin, causing the drape to peel from the skin over time.

In this study, the amount of skin traction caused by the surgical technique appeared to be similar in both groups. However, the prototype drape with the flexible base film was more resistant to peeling from the surgical edge because it had better tissue conformability to the deformation of the edge caused by the surgeon’s manipulation. In addition, it has been reported that plastic drapes were more likely to peel off when the skin was highly moist (34). As described above, in the existing drapes, there was more peeling between the skin and the drape immediately after skin incision, which might have allowed more moisture to penetrate between the skin and the drape during surgery. Therefore, it was thought that in the existing drape group, the adhesive strength between the drape and the skin decreased over the course of surgery, resulting in a greater difference between the two groups before skin suturing than immediately after the skin incision. It has also been reported that moisture penetrating between the skin and the drape could dilute the disinfectant impregnated in the drape, potentially reducing the effectiveness of the disinfectant (34). Therefore, it is desirable to keep the moisture between the skin and the drape as small as possible.

This study was conducted in cat contraceptive surgery, which is one of the most common surgeries in veterinary medicine. However, it is not known whether the results of this study apply to all surgical procedures and all ages for dogs and cats. This is because the elasticity of the skin, the amount of sebum, and the degree of dehydration vary depending on age, sex, breed, and environment, and these factors have a significant effect on the adhesive strength of the skin (35–37). In addition, other surgical techniques may have different traction forces in the surgical field, the amount of fluid such as exudating and bleeding, and operative times, so the results of this study may not be representative of various surgical techniques. Therefore, further studies on different animal species and surgical procedures are needed to confirm the results of this study.

Furthermore, details regarding the materials and manufacturing methods of plastic drapes are not publicly disclosed by each company. Therefore, since the prototype drapes appear different from existing drapes, surgeon bias is unavoidable in comparative studies of drapes.

This study targeted non-specialist surgeons in general clinics who were unfamiliar with conducting research. Therefore, a simplified gradation scale was used to assess the degree of drape peeling conducted by the surgeon. However, to eliminate surgeon subjectivity, it might have been ideal to measure the degree of drape detachment using a scale or similar method rather than a gradation scale.

We also acknowledge that the study was conducted across five clinics and that center/surgeon effects could not be reliably estimated because of the limited number of cases per center.

The results showed that the final peeling area was largely dependent on that immediately after skin incision. This study concluded that the use of flexible plastic drapes with a base film with a low Young’s modulus reduced drape peeling during surgery compared to the existing drapes used in neutering surgery of cats. In recent years, an increasing number of research reports have suggested the usefulness of drapes in preventing infections, as well as a correlation between drape lifting and infection risk. In other words, the need for drapes that are difficult to peel off during surgery has increased significantly. As the results of this study suggest, improving the structural condition of the drape can reduce intraoperative drape peeling and contribute to improving surgical outcomes.

## Data Availability

The raw data supporting the conclusions of this article will be made available by the authors, without undue reservation.
